# Crescentic glomerulonephritis due to linear IgA anti-glomerular basement membrane disease: report of a rare case

**DOI:** 10.1590/1414-431X2024e13466

**Published:** 2024-05-03

**Authors:** A.G. Monich, R.F. Romani, J.L.S. Carneiro

**Affiliations:** 1 Serviço de Nefrologia, Hospital Universitário Evangélico Mackenzie, Curitiba, PR, Brasil; 2 Disciplina de Nefrologia, Faculdade Evangélica Mackenzie do Paraná, Curitiba, PR, Brasil

**Keywords:** Anti-glomerular basement membrane disease, Immunoglobulin A, Renal insufficiency, Chronic

## Abstract

Anti-glomerular basement membrane (GBM) disease is a rare and severe vasculitis that affects the glomerular and pulmonary capillaries and has an incidence of less than 2 cases per million individuals per year. Anti-GBM disease is mediated by autoantibodies against the α3 chain of type IV collagen. In the majority of cases, the autoantibodies are of the immunoglobulin G (IgG) class, with rare cases being mediated by immunoglobulin M (IgM) or immunoglobulin A (IgA); there are less than 15 IgA-mediated cases reported in the literature worldwide. The classic form of this disease manifests with rapidly progressive glomerulonephritis (RPGN), with or without pulmonary hemorrhage, and the diagnosis consists of identifying high titers of autoantibodies in the serum and/or deposited in the tissues. IgA antibodies are not identified in routine immunoassay tests, and renal biopsy with immunofluorescence is essential for diagnosis. We present a case of RPGN due to anti-GBM disease with linear IgA deposition, whose diagnosis was made exclusively by renal biopsy and with an unfavorable prognosis.

## Introduction

Anti-glomerular basement membrane disease (anti-GBM) is a rare small vessel vasculitis that mainly affects glomerular and pulmonary capillaries and may affect capillaries in the choroid plexus and cochlea to a lesser extent. The eponym “Goodpasture Syndrome” is also used to describe anti-GBM disease when it is characterized by pulmonary hemorrhage associated with rapidly progressive glomerulonephritis (RPGN). Due to its rarity, the incidence of the disease is uncertain; studies point to an incidence of less than 1 case/million inhabitants/year in Europe, and it is even rarer in African populations (1). It shows a bimodal age distribution, with peaks in incidence in the third and sixth decades; in younger patients, there is a slight predominance of cases in men and pulmonary involvement is more frequent, while there is a predominance of cases in women and more cases of isolated glomerulonephritis in the older population (1-5).

In the pathophysiology of the disease, autoantibodies against specific antigens of the glomerular basement membrane are involved; in most cases, the autoantibodies are polyclonal immunoglobulin G (IgG), with rare cases being mediated by autoantibodies polyclonal immunoglobulin A (IgA), polyclonal immunoglobulin M (IgM), or gammopathy-associated monoclonal immunoglobulin (6). Less than 15 cases of anti-GBM disease due to IgA deposition have been described in the literature, and no cases have been reported in Brazil (2,7-9).

The classic form of anti-GBM disease manifests with nephritic syndrome and RPGN, with or without pulmonary hemorrhage. Approximately 80-90% of patients have RPGN, 40-60% have concomitant pulmonary hemorrhage, and a small minority of patients have isolated lung disease (approximately 5%) (1,5,10).

Although anti-GBM disease is uncommon, it accounts for 10-20% of all cases of RPGN in renal biopsies. Characteristically, the glomerular crescents have the same evolutionary age and affect approximately 75% of the sampled glomeruli (1,7). Immunofluorescence microscopy shows a strong linear deposition of autoantibodies in the GBM, classically of the IgG type. There may be deposits in the GBM of C3 to a lesser extent and deposits in the tubular basement membrane due to antibody cross-reactivity (1,2,8,11).

The diagnosis of anti-GBM disease consists of identifying the pathogenic autoantibodies, either in serum and/or deposited in the tissues. Immunoassay serological tests may not detect IgA class anti-GBM antibodies, emphasizing the importance of renal biopsy in diagnosing the pathology (1-3,5,7,12,13).

The treatment of RPGN due to anti-GBM disease consists of immunosuppression with corticosteroids and cyclophosphamide, and plasmapheresis can also be used (1,2). Renal prognosis correlates with the severity of the disease at presentation and can progress rapidly to end-stage renal disease, if left untreated. The predictors of worse renal outcomes include the severity of renal dysfunction at disease presentation, especially if the serum creatinine level ≥5.7 mg/dL (503.9 μmol/L), the ratio of glomeruli affected by crescents, and oligoanuria at presentation. Kidney transplantation is a therapeutic option if anti-GBM antibodies are negative for at least 6 months (1-3,7,8,14).

Here, we report a case of RPGN due to anti-GBM disease with linear IgA deposits in renal biopsy, with a negative immunoassay serological test and an unfavorable renal outcome. This report was approved by the Research Ethics Committee of the Instituto Presbiteriano Mackenzie (Brazil).

## Case report

A 41-year-old female patient was admitted to the nephrology department due to edema of the lower limbs and periorbital area for 20 days, with no respiratory symptoms. The patient denied any previous illnesses, did not use medication, or had relevant family history. No prior laboratory tests were available for comparison.

Upon physical examination, the patient presented with edema of the lower limbs and skin pallor, and the blood pressure was 140/80 mmHg.

On admission, the serum creatinine value was 7.8 mg/dL (689.54 µmol/L), with anemia (hemoglobin 6.5 g/dL), hypoalbuminemia (serum albumin 3.0 g/dL), dysmorphic hematuria, and proteinuria at nephrotic levels (6.8 g/day). An ultrasound showed normal-sized kidneys without loss of corticomedullary differentiation and a chest x-ray without evidence of pulmonary hemorrhage. Laboratory tests revealed negative antineutrophil cytoplasmic antibodies and negative antinuclear antibody, as well as normal levels of complement fractions C3 and C4. An immunoassay test for IgG class anti-GBM antibodies was negative and no monoclonal band was detected in serum protein electrophoresis. Tests for viral hepatitis and human immunodeficiency virus (HIV) were also negative.

Immunosuppression was started with intravenous 1 g/day of methylprednisolone for 3 days and a kidney biopsy was carried out. During hospitalization, there was no clinical evidence of pulmonary hemorrhage and kidney function did not improve.

**Figure 1:**
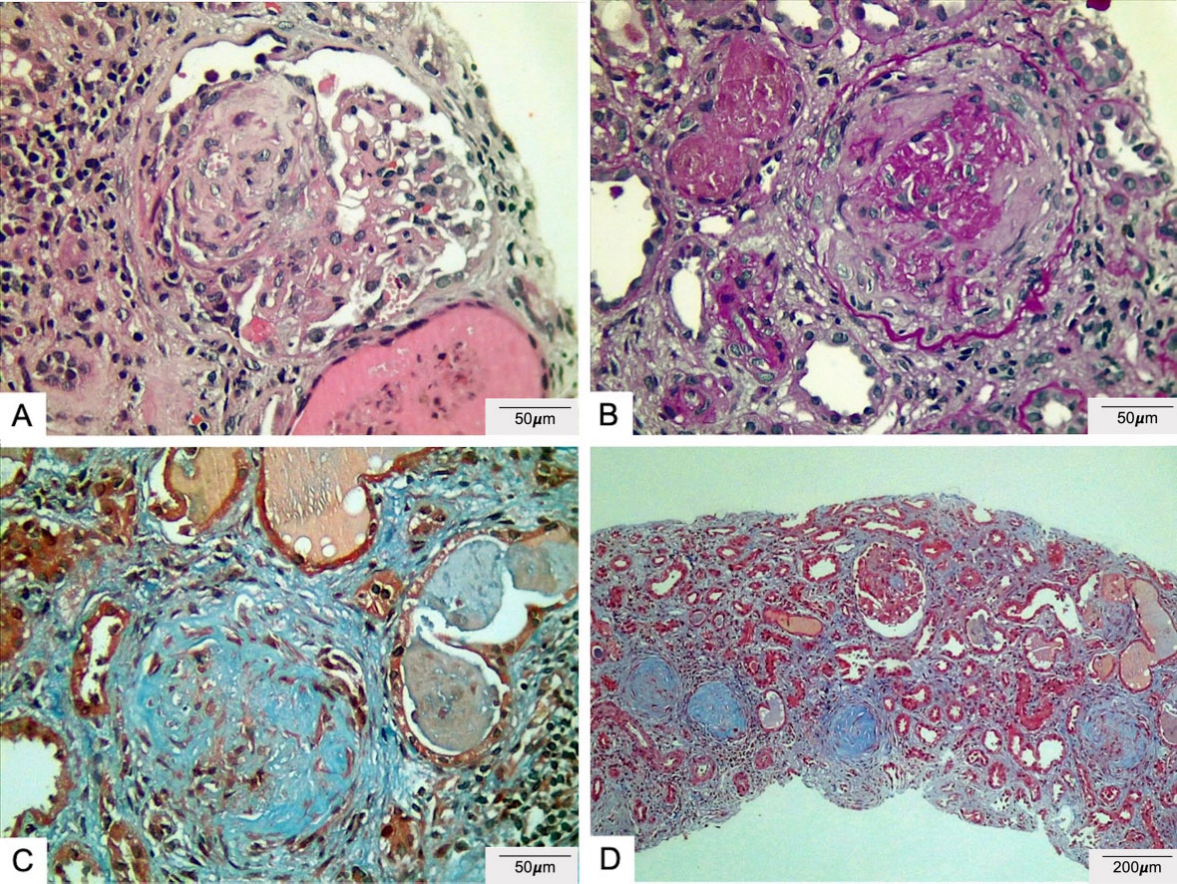
Light microscopy. The sample contained 21 glomeruli and fourteen (67%) showed global sclerosis. All the glomeruli are surrounded by crescents. The interstitial space is enlarged by marked fibrosis. **A**, hematoxylin-eosin; **B**, periodic acid-Schiff; **C** and **D**, trichrome. Scale bar 50 μm (**A**-**C**) and 200 μm (**D**).

**Figure 2:**
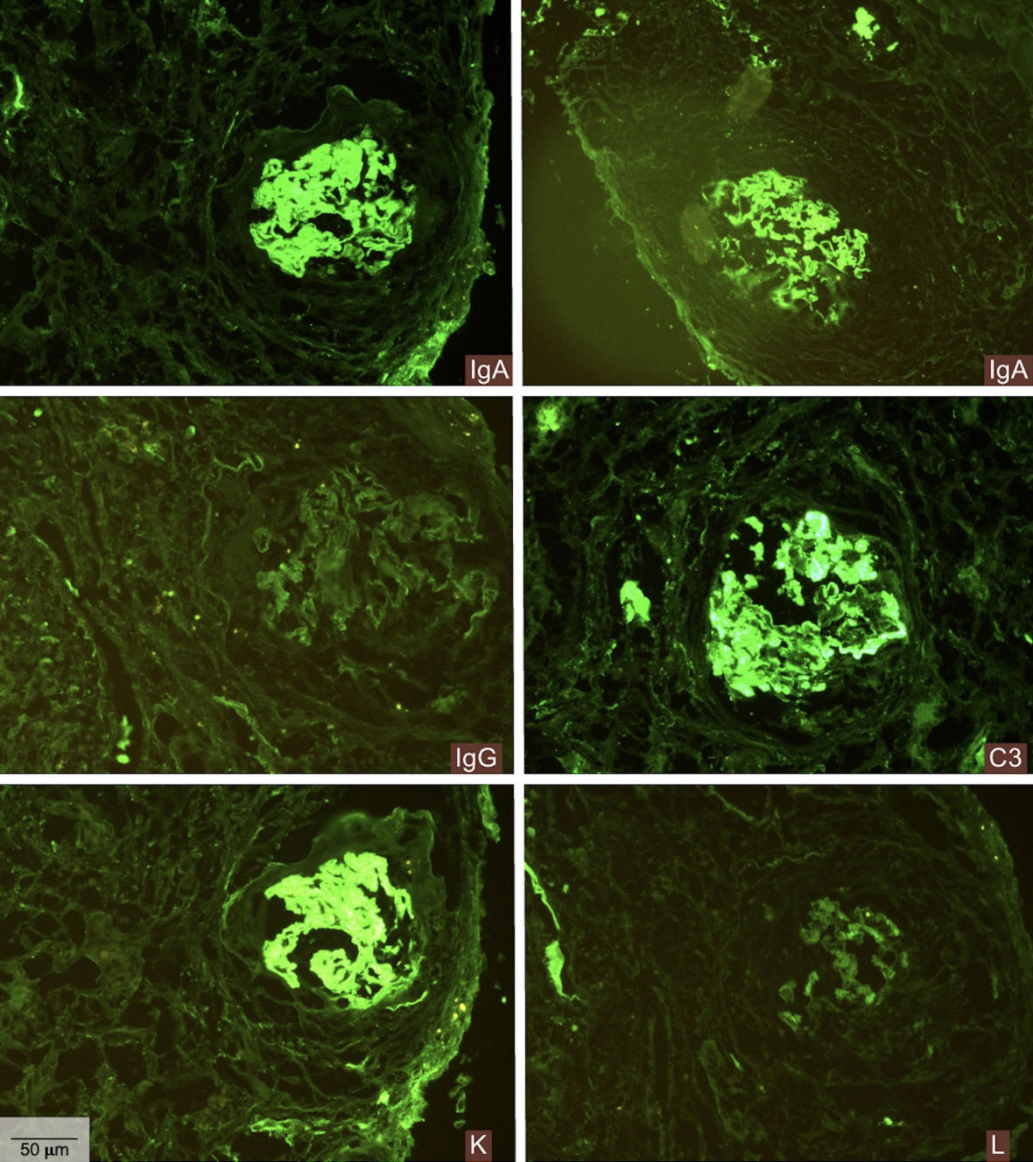
Immunofluorescence microscopy. A positive linear pattern of strong intensity for IgA in the capillary loops was observed along the glomerular basement membrane. Strong immunolabeling of the same pattern is associated with C3, kappa (K), and lambda (L). IgA, kappa, and lambda were also positive and linear in tubular membrane and Bowman's capsule. The other markers, including IgG, fibrinogen, and C1q were negative. Scale bar 50 μm.

The renal biopsy sample contained 21 glomeruli: 14 (67%) of these showed global sclerosis, 19 (90.4%) were enveloped by fibrous crescentic lesions, and two by fibrocellular crescents. Furthermore, marked interstitial fibrosis (70-80%) and an interstitial mononuclear inflammatory infiltrate, as well as frequent red cell casts and foam cells filling the tubules were observed (Figure 1). Immunofluorescence microscopy revealed a strong linear pattern of IgA positivity in the capillary loops along the glomerular basement membrane, as well as in tubular membranes and Bowman's capsule. IgG labeling was negative. Kappa staining was positive and with a linear pattern in capillary loops along the glomerular basement membrane, in tubular membranes, and Bowman's capsule; lambda staining was also positive, with linear pattern in capillary loops along the glomerular basement membrane (Figure 2). Light microscopy findings associated with the immunofluorescence showed rapidly progressive crescentic glomerulonephritis, an immune-mediated form against the GBM, described as anti-GBM disease due to IgA class autoantibodies.

Due to the existence of stigmata of chronic kidney disease with significant global sclerosis of the glomeruli on biopsy, extensive glomerular involvement by fibrous crescents, and marked interstitial fibrosis, immunosuppression was suspended, and the patient was referred for renal replacement therapy with an estimated glomerular filtration rate of 5.3 mL·min·1.73 m^2^ according to the Chronic Kidney Disease Epidemiology Collaboration.

## Discussion

The first case series of anti-GBM disease was described in 1958, and recent studies indicate that the disease manifests after alterations in the collagen structure, leading to exposure of the antigen allowing interaction with circulating anti-GBM antibodies (9). There is a strong genetic predisposition associated with HLA-DR15; however, some environmental factors are also implicated in the pathogenesis of the disease by acting as triggers, in particular exposure to smoking, hydrocarbons, infections, lithotripsy, alemtuzumab, and infections by Gram-negative bacteria (1,7,9). The disease is mediated by autoantibodies (usually polyclonal IgG class) against specific antigens of the GBM, the main target being the non-collagenous domain (NC1) of the α3 chain of type IV collagen, also known as the “Goodpasture antigen” (1,2,6,7). In IgA-mediated disease, antibodies appear to target the α1 and α2 chains of type IV collagen (2,7,8).

It is supposed that the exposure to these environmental triggers can modified the structure of type IV collagen and expose cryptic epitopes to the immune system (15). In addition to autoantibodies, macrophages and CD4(+) T cells are present in the glomeruli of mice with anti-GBM disease. Some cell-mediated and antibody-independent mechanisms were tested in animal models, and it appears that T cells regulate the anti-α3(IV) NC1 antibodies and are effectors of inflammatory response, playing a role in the pathogenesis of glomerulonephritis (15,16).

Although the most common pathogenic autoantibodies in anti-GBM disease are of the IgG class (with IgG1 and IgG3 predominating), rare cases of disease mediated by IgA and IgG4 class antibodies have been described. Immunoassay tests are more specific and more sensitive for IgG1 and IgG3 once they detect antibodies against α3(IV)NC1. In cases of atypical anti-GBM disease with IgA, IgM, or IgG4 antibodies with other target antigens, the immunoassay tests may be falsely negative (17). Therefore, immunofluorescence of the kidney biopsy is essential for diagnosis, since up to 10% of patients can have negative serum tests (1,8), as seen in the reported case. The distribution pattern of Ig-A antibodies in immunofluorescence with positivity in the capillary loops along the glomerular basement membrane, tubular membrane, and Bowman's capsule reflects the distribution of the α1/α2(IV) collagen chains, which are the targets of the antibodies in this atypical presentation of anti-GBM disease (7,8). The use of renal biopsy contributes to both the diagnosis and prognosis of the disease; however, when there is a strong suspicion of RPGN, biopsy should be indicated and carried out as soon as possible, and specific treatment instituted regardless of whether biopsy is carried out (18).

The introduction of plasmapheresis in the 1970s changed the prognosis of patients with pulmonary hemorrhage and kidney disease in the early stages. When used early in combination with immunosuppressive therapy, it can prevent progression to end-stage renal disease and improve outcomes (19,20). In addition to plasmapheresis not being available in several health institutions in Brazil, in the reported case the lungs were not involved and the kidney damage was already chronic, so plasmapheresis was not carried out in this patient.

Immunosuppressive therapy is questionable for those patients who have worse prognostic factors due to the risks inherent to immunosuppression (14); in the case presented in this report, both the severity of the kidney damage on admission and the high prevalence of fibrous glomerular growths in the biopsy led to the suspension of immunosuppression, corroborating published reports (1,7,14).

Compared to IgG-mediated anti-GBM disease, the prognosis of IgA-mediated disease is poor and most cases progress to end-stage renal disease (2,3). Of 14 cases reported in the literature, 7 patients required long-term renal replacement therapy and 5 patients remained with severe chronic kidney disease (2,3,8). Kidney transplant in IgA anti-GBM disease could be challenging since the immunoassay tests could be negative with no possibility of monitoring the presence of circulating autoantibodies (1-3,8,10). There are related cases with alternative laboratory tests such as immunoblotting and modified indirect immunofluorescence that were used to confirm the diagnosis and to authorize and follow-up the transplantation (8).

We described a rare variation of anti-MBG disease due to IgA-type autoantibodies, in which renal biopsy with immunofluorescence was a fundamental diagnostic tool, given the negative serum IgG-type anti-GBM antibody in these cases, providing important data on the chronicity of the lesions and renal prognosis.
